# An empirical analysis of the impact of income inequality and social capital on physical and mental health - take China’s micro-database analysis as an example

**DOI:** 10.1186/s12939-021-01560-w

**Published:** 2021-11-06

**Authors:** Yuanyuan He, Lulin Zhou, Junshan Li, Jun Wu

**Affiliations:** 1 School of Management, Jiangsu University, Zhenjiang, 212002 Jiangsu China; 2 School of Economics and Management, Jiangxi University of Chinese Medicine, Nanchang, 330000 Jiangxi China

**Keywords:** Income inequality, Kakwani index, Social capital, Physical and mental health, Intermediary effect

## Abstract

**Background:**

Income inequality is one of the important reflections of the unbalanced development of the world economy and can have adverse effects on physical and mental health.

**Methods:**

This article used the 2018 China Family Panel Studies Database as an empirical analysis data source. The Kakwani index (KI) was used to measure income inequality, and social capital was broken into cognitive social capital and structural social capital. Our assessment was conducted by using STATA16 software for ordered logistic regression, verifying income inequality, social capital on correlation between physical and mental health firstly; then by gradual regression methods to verify intermediary effect, and demonstrate the social capital as an intermediary variable affecting physical and mental health as income inequality.

**Result:**

The income inequality has a significant negative effect on physical and mental health (β = − 0.964, − 0.381; OR = 0.382, 0.758; *P* < 0.01), Social capital has a significant effect on physical and mental health (Cognitive SC(MH): β = 0.146 and 0.104, OR = 1.157 and 1.110, *P* < 0.01; Cognitive SC(PH): β = 0.046 and 0.069, OR = 1.047 and 1.071, *P* < 0.01; Structural SC(MH): β = − 0.005, 0.025 and 0.015, OR = 0.995, 1.025 and 1.015, *P* > 0.1, *P* < 0.01 and *P* < 0.01; Structural SC(PH): β = − 0.026, 0.009 and − 0.013, OR = 0.975, 1.009 and 0.987, *P* < 0.01, *P* > 0.1 and *P* < 0.01). Our analysis also showed that social capital (cognitive social capital and structural social capital) has an intermediary effect on physical and mental health due to income inequality.

**Conclusion:**

This study shows that income inequality can not only directly affect physical and mental health, but also through social capital intermediary utility indirectly affect physical and mental health, social capital has positive effects on physical and mental health. At the same time, income inequality and social capital’s effects on physical and mental health exist regional differences, urban-rural differences, and gender differences. Therefore, in the development of special policies to support and take care of vulnerable groups, special attention needs to be paid to poor rural areas and female groups.

**Supplementary Information:**

The online version contains supplementary material available at 10.1186/s12939-021-01560-w.

## Introduction

According to the 2020 World Social Report of the Department of Economic and Social Affairs of the United Nations, issues of inequality remain endemic in both developed- and developing- countries. The World Health Organization estimates that about 70% of the world’s population still lives in inequality-exacerbated countries, and the level of inequality is in a historic high today. Among them, income inequality (IIequality) is the main problem, which is the reflection of unbalanced economic development [1, 2]. Although the IIequality between countries has been reduced, this issue is getting increasingly serious, especially in developing countries [3]. China is no exception and IIequality is one of the main reasons why health revenue stagnation, resulting in decreased human life by 0.4 years for women and 0.6 years for men [4].

The path that IIequality effects on physical and mental health (PH and MH) is divided into direct path and indirect two ways [5]. The analysis of the impact mechanism of income affecting health mainly revolves around the dimensions of absolute income, relative income and income deprivation [6]. First, the IIequality can directly act on individual PH and MH, resulting in increased mortality [7], shortening per capita life [8], affecting the physiological functions [9] and MH [10]. Second, IIequality can be indirectly active in PH and MH through eroding social capital (SC) [11]. Although more and more evidence suggests that IIequality is related to PH and MH, there is still disagreement in the research conclusions of IIequality and PH and MH [12]. A more in-depth assessment is needed in the future. Especially, current researches on IIequality are concentrated more in the group level [13, 14] and national level [7, 15], but relatively few at a personal level.

Social capital can affect both the IIequality and PH&MH of an individual. Regarding, IIequality, SC plays a double role. Firstly, it can enhance personal endowment, increase income, improve social status through its poverty reduction effect and stabilization effect, and improve IIequality [16]. On the other hand, SC can weaken capital liquidity by influencing investment behavior and capital transmission paths, leading to continued widening of the income gap [17]. Secondly, SC has an impact on PH and MH. SC can directly affect PH and MH through social equity, social trust, and social network density which can in turn positively impact on an individual’s self-rated health and self-rated MH. Further, the extant literature also presents a plethora of studies that support that SC can significantly reduce mortality, suicide rate, and alleviate depression [18–20]. Moreover, SC can affect a person’s health by affecting medical services utilization. In the work of Zhao et al. (2019), it is observed that different types of SC can have different effects on health [21, 22]. Even though more and more scholars are concerned about the impact of SC on IIequality and PH and MH, there are few studies on the synergy of the three elements [23, 24]. Specifically, there is a paucity of studies that systematically demonstrate the intermediary role of SC in the relationship between IIequality and health (PH and MH).

Regression analysis has been the common method currently used to assess the relationship between IIequality, SC and PH and MH [25], with more focus on adolescents [24] and the elderly [11] in developed countries. There is a lack of relevant arguments for developing countries. This study selected China as the research object, which is the largest developing country. The data source is the results of China Family Panel Studies (CFPS) in 2018. This is advantageous to validating the interplay between IIequality, SC and health because 2018 CFPS is the single largest source of data pertaining to the SC, health, and IIequality and other related measurement indicators for one country. The standard estimate of “household income per capita” and “individual income” for 2018 as revealed by the CFPS indicates that China’s Gini coefficients are 0.508 and 0.477, respectively, which are both higher than the 0.4 warning level. This indicates that the current income gap in China is excessively serious, thus requiring immediate attention to resolve to avert further exacerbation. Navigating the reason why China’s persistent and resilient economic development has not translated into total eradication of a large tract of economic disparity is of academic interest. The study then applies ordered logistic regression and other methods to do robustness tests to study the impact of IIequality and SC on PH and MH. Subsequently, the KHB (Karlson-Holm-Breen) method is applied to test the mediating effect of SC in the path of IIequality on PH and MH. The findings of this study will have important enlightenment for developing countries to solve social problems caused by IIequality.

## Research design and method section

### Data source and processing

The research data came from China’s 2018 CFPS database, which is a national social survey project with a two-year survey cycle, including individual, family, community, and other levels of survey data. The survey covered 31 provinces/cities/autonomous regions of China including 14 in Eastern China, 6 in Central China, and 11 in Western China covering nearly 95% of the regional population. It is a typical and representative micro-database in China with more than 30, 000 interviewees. The study selected the content of family questionnaires and adult questionnaires as the main data sources (*N* = 33,326), and eliminated invalid data such as “null values, refusal to answer or don’t know”. The effective sample size is 22, 751, women accounted for 52.21% and men accounted for 47.79%, rural population was 50.75% and urban population was 49.25%, the average age of the sample was 47.76, and the average years of education was 7.325 years. The proportion of participating in basic medical insurance is 91.6% (including medical insurance for urban employees and urban residents). Because the data source is cross-sectional, a multicollinearity test for verifying variance inflation factor and Tolerance were performed to avoid the linear correlation of the explanatory variables from affecting the regression results. Except for the “region” variable, the variance inflation factor value of other analysis variables are all less than 2, and Tolerance values are all more than 0.5.

### Method section and model construction

Because the assignments of variables related to PH and MH are ordinal categorical variables, the ordered logistic regression model was used for model estimation. The measurement model is as follows:

1 $${Health}_i^{\ast }=\beta {X}_i+{prov}_i+{\mu}_i+{\varepsilon}_i\left(i=1,2,\sim n\right)$$

The variable *Health*_*i*_, refers PH and MH, they are unobservable latent variables; IIequality, SC and other CVs are all included in the vector *X*_*i*_; μ_i_ is the individual effect; and *prov*_*i*_ refers to province fixed effect; *ε*_*i*_ is a random error term that obeys a logical distribution, the individual selection rule is:

2 $${Health}_i^{\ast }=\left\{\begin{array}{l}1, if\kern0.5em \left({Health}_i^{\ast}\le {r}_0\right)\\ {}2, if\kern0.5em \left({r}_0\le {Health}_i^{\ast}\le {r}_1\right)\\ {}3, if\kern0.5em \left({r}_1\le {Health}_i^{\ast}\le {r}_2\right)\\ {}4, if\kern0.5em \left({r}_2\le {Health}_i^{\ast}\le {r}_3\right)\\ {}5, if\kern0.5em \left({r}_3\le {Health}_i^{\ast}\le {r}_4\right)\end{array}\right.$$

Among them, *r*_*0*_ *< r*_*1*_ *< r*_*2*_ *< r*_*3*_ *< r*_*4*_, they are called the cutoff points, *ε*_*i*_ obeys the logical distribution, F(*φ*) = exp.(*φ*)/[1 + exp.(*φ*)], so the probability distribution function is expressed as:

3 $${\displaystyle \begin{array}{c}P=\left({Health}_i=1\left|{X}_i,\beta, {prov}_i,{\mu}_i\right.\right)=P\left({Health}_i^{\ast}\le {r}_0\left|{X}_i,\beta, {prov}_i,{\mu}_i\right.\right)=P\left(\beta {X}_i+{prov}_i+{\mu}_i+{\varepsilon}_i\le {r}_0\left|{X}_i,\beta, {\mu}_i\right.\right)\\ {}=P\left({\varepsilon}_i\le {r}_0-{X}_i\beta -{\mu}_i-{prov}_i\left|{X}_i,\beta, {\mu}_i\right.\right)=F\left({r}_0-{X}_i\beta -{\mu}_i-{prov}_i\right)\end{array}}$$

4 $$P=\left({Health}_i=2\left|{X}_i,\beta, {prov}_i,{\mu}_i\right.\right)=F\left({r}_1-{X}_i\beta -{\mu}_i-{prov}_i\right)-F\left({r}_0-{X}_i\beta -{\mu}_i-{prov}_i\right)$$

5 $$P=\left({Health}_i=3\left|{X}_i,\beta, {prov}_i,{\mu}_i\right.\right)=F\left({r}_2-{X}_i\beta -{\mu}_i-{prov}_i\right)-F\left({r}_1-{X}_i\beta -{\mu}_i-{prov}_i\right)$$

6 $$P=\left({Health}_i=4\left|{X}_i,\beta, {prov}_i,{\mu}_i\right.\right)=1-F\left({r}_3-{X}_i\beta -{\mu}_i-{prov}_i\right)$$

IIequality may erode SC to a certain extent. To further assess the mediating role of SC in the path of IIequality affecting PH and MH, the study adopted the KHB approach [26–28] to test the mediating utility of SC and constructed a mediating effect regression model. The KHB analysis method is suitable for estimating the intermediary variables of the nonlinear probability models. It can not only analyze the binary logistic regression model but also can be used to analyze the ordered multi-class logistic regression. The equation of the model is shown in the formulas 7, 8, 9 below:

7 $${Health}_i^{\ast }={\beta}_0+{aIInquality}_i+\gamma {X}_i+{prov}_i+{\mu}_i+{\varepsilon}_i$$

8 $${SC}_i={\beta}_1+{bIInquality}_i+\gamma {X}_i+{prov}_i+{\mu}_i+{\varepsilon}_i$$

9 $${Health}_i^{\ast }={\beta}_2+{a}^{\prime }{IInquality}_i+{cSC}_i+\gamma {X}_i+{\mu}_i+{prov}_i+{\varepsilon}_i$$

Formula (7) tests the total effect of IIequality on health which is a “simplified model”, formula (8) tests the correlation between IIequality and SC, and formula (9) tests the correlation coefficients of independent variables after controlling SC which is a “full model”; *Health*_*i*_ are unobservable latent variables whose formulas are represented as (3)–(6), the Direct effect (DE), and Total effect (TE) are shown in the formula (10). Among them, *σ*_*b*_ and σ_a_ are the scale parameters, which are derived from the standard error of the residuals of the linear regression Eqs. (7) and (9), and *σ*_*a*_ ≤ *σ*_*b*_. Therefore, the measurement method of the Intermediary effect (IE) in the logistic model is shown in Eq. (11). The specific measurement results can be completed by STATA16 software.

10 $$DE=\frac{a}{\sigma_a}, TE=\frac{b}{\sigma_b}$$

11 $$IE= TE- DE=\frac{b}{\sigma_b}-\frac{a}{\sigma_a}$$

### Variable selection

#### Dependent variable (DV)

The health variables of regression analysis include two dimensions: PH and MH. Physical health refers to the development cycle and severity of the disease as a whole and adopts “whether the body feels unwell in the past two weeks”, “whether there is any chronic disease within six months”, “have you seen a doctor”, and “whether the cause is caused in the past 12 months”. The above four indicators mainly measure individual PH by illness and hospitalization, if the answer of the indicator is “yes”, it is assigned “0”, if the answer is “no”, it is assigned “1”. According to the total score, four points of PH level 1–4 are obtained. Mental health is based on the eight psychological survey questions QN406-QN420 in the “Behavior and Mental State” module of the questionnaire, which mainly includes indicators such as personal sleep status and loneliness. According to the directional content of the indicators, conduct 1–4 Corresponding points. The total score of MH status is 8–32 points.

#### Independent variable (IV)

The dependent variable includes two variables, namely IIequality and SC.

##### Income inequality (IIequality)

The study used the individual income deprivation index (Kakwani index, KI) as a measure of IIequality at the individual level [5, 29], which emphasizes that IIequality at the individual level comes from the comparison of individuals with high-income reference groups. Commonly used individual deprivation indexes are Yitzhaki index [30], KI [31] and Podder index [32]. The KI is the development of the Yitzhaki index, which overcomes the latter’s sensitivity to changes in income scale [33], and at the same time makes up for the shortcomings of indecomposability and non-addition of the Gini coefficient. The relative deprivation of group members due to income gap can be observed objectively, and the IIequality at the individual level can be visualized. Therefore, this study used the KI to measure IIequality and the specific calculation formula is as follows:

12 $$RD\left(I,{I}_i\right)=\frac{1}{n{\mu}_I}\left[\sum \limits_{j=i+1}^n\left({I}_j-{I}_i\right)\right]={\gamma}_{I_i}^{+}\left[\frac{\left({\mu}_{I_i}^{+}-{I}_i\right)}{\mu_I}\right]$$

Of which, household per capita income was used to calculate the individual income deprivation index. I is the reference income group composed of all the variables filtered in the CFPS database, the number of groups is n, and the income of individuals in the reference group is arranged in ascending order to form an income vector set (*I*_*1*_*, I*_*2*_*, I*_*3*_*, ..., I*_*n*_). In formula (12), μ_I_ represents the average income of all samples in group I, ${\gamma}_{I_i}^{+}$ is the proportion of the number of samples in I group whose income exceeds Ii in the total sample volume of heatstroke, and ${\mu}_{I_i}^{+}$ is the average of sample incomes in group I with income exceeding Ii. In the heterogeneity test, due to changes in the characteristics of the reference group, the vector set of the reference group will also change accordingly. The corresponding reference group income deprivation index was recalculated using the same method described above.

##### Social capital (SC)

Social capital is a characteristic of social organizations, and it is the product of the fusion of subjective social norms and objective social characteristics. Among them, the organizational network and the mutual trust and reciprocity among members of the organization can strengthen the cooperative relationship between individual members, thereby promoting the efficiency of the organization and society. Improve [34]. Social capital is divided into cognitive and structural SC [35]. Cognitive SC refers to the mutual trust and mutual assistance between members of social organizations, which are measured by indicators of social justice and social trust. Structural SC refers to the network of relationships among members of an organization, which is measured basing on social support, relationship, and network density. First of all, the sense of social justice used N601 “How do you think the following problems are in our country” to comprehensively calculate scores, including housing, medical care, social security, wealth gap and other social issues. Secondly, the social trust was calculated by using a comprehensive score for the trust of parents, relatives, neighbors, strangers and government officials; Social support used the sum of social funds given by children, relatives and others and aid funds given to others by oneself as a measurement indicator; Personal relationship is closely related to the social resources that individuals can mobilize [36], so structural SC was included for targeted analysis, and QM2011 “How good is your relationship” question was used as the research indicator. Finally, China is a traditional relational society, and etiquette, obedience, and human relationship are important ways to maintain social networks and interpersonal relationships [37], so the FU201 “Relationship and Gift Expenditure Measurement” was used to measure the density of the relationship network [38, 39].

#### Covariates

Considering the impact of other factors on PH and MH and minimizing indigenousness and heteroscedasticity, this study incorporated the individual’s characteristics (ICV), social characteristics (SCV), living habits, and medical environment (ME) into the analysis framework: a) Taking household registration, gender, age, marital status, years of education, region as ICVs; b) Working status, insurance participation, family size, social status, etc. were selected as SCVs; c) Smoking, drinking, exercise, lunch break, etc. were selected as descriptive variables for indications of lifestyle habits; and d) the patient’s satisfaction with the diagnosis and treatment environment and the diagnosis and treatment level were considered as the ME. The basic description and statistical results of the variables are shown in Table 1.

**Table 1:** Variable description and statistics

**Type**	**Name**	**Description**	**Mean**	**SD**
**Health Variable**	Xlhealth	MH, score range: 1–5, the large score, the healthier	4.239	0.818
	Kghealth	PH, score range: 1–4, the large score, the healthier	3.378	0.845
**IIequality**	Kakwani	calculated using KI, value is 0–1	0.508	0.246
**SC**	Trust	Average value of trust, ranges 0–10, large value means higher trust	5.575	1.343
	Fair	Average value of fairness, score ranges 1–8, fairness score from low to high	3.309	1.540
	Support	Social support, total reciprocal financial assistance, logarithm	4.220	3.983
	Relations	Relationship level: 0–10 level increasing	7.109	1.981
	Jiegousc	Network density, measured by expense of favors gifts, logarithm	7.277	2.437
**ICV**	Urban	Household registration, urban = 1, rural = 0	0.493	0.500
	Gender	Gender: male = 1, female = 0	0.478	0.500
	Age	Age, actual age, value ranges: 20–81	47.77	15.44
	Marriage	Marital status, with spouse = 1, without spouse = 0	0.825	0.380
	Education	Years of education, from illiterate to doctorate, value range: 0–22	7.331	4.920
**SCV**	Employ	Working status, employed = 1, unemployed = 0	0.777	0.416
	Insurance	Insurance participation, Is there social medical insurance? Yes = 1, No = 0	0.916	0.277
	Family	Family size, number of family members, value range: 1–21	4.145	2.081
	Status	Social status, range: 1–5, mean from low to high	3.103	1.092
**Living habit**	Exercise	Exercise, the frequency of physical exercise in last week. Value is 0–50	2.496	3.295
	Smoke	Smoking: Smoking in the past month? Yes = 1, No = 0	0.299	0.458
	Drink	Drinking: 3 times per week in the past month? Yes = 1, No = 0	0.155	0.362
	Break	Lunch break, Yes = 1, No = 0	0.536	0.499
**ME**	Satisfaction	Medical condition, score range: 1–5, means dissatisfied to satisfied	3.628	0.814
	Care	Medical level, score range: 1–5, from very bad to very good	3.499	0.885

*SD* Standard deviation, *IIequality* Income inequality, *SC* Social capital, *ICV* Individual characteristic variables, *SCV* Social characteristic variables, *ME* Medical Environment, *MH* Mental health, *PH* Physical health, *KI* Kakwani index

### Robustness test

This study conducted a variety of methods for robustness testing to ensure the science of research methods and research indicators and the reliability of research conclusions. At first, replace the index of the DV was replaced by taking self-rated health data as the explained variable and estimated the Ordered Logistic Regression according to the variable type as the ordered multi-category discrete variable. The test results are shown in Table 4 (1). Secondly, the IV form and the KI with Podder index were replaced by taking the logarithm of the family’s per capita income and then calculate it according to the Yitzhaki index measurement method. The test results are shown in Table 4 columns (2) and (3). At the same time, in order to eliminate the influence of outliers on the regression results, the Winsor shrinking method was used to process the samples up and down 1% of the statistical indicators, and the remaining 22,296 samples were re-regressed. Finally, the test model was changed to convert the MH and PH data into continuous numerical variables. The total score of MH status is 8–32 points, and four points of PH are obtained, then the ordinary least squares regression was used for statistical analysis.

### Heterogeneity validation

In the analysis of the influence of the Covariates on the DV, it is found that both household registration and gender will have a significant impact on PH and MH. It is speculated that there may also be gender differences and household registration differences in the effect of IIequality and SC on the DV, therefore, gender and household registration are selected as the dimensions of the heterogeneity validation. In addition, the eastern, central, and western parts of China show a gradual decline in income levels, so regional variables are also used as one of the dimensions of heterogeneity validation.

## Results

### Benchmark regression results

As shown in Tables 2 and 3, Benchmark regression included two main parts: the first is the independent regression of the IV that controls other key variables, and the second is the integrated regression that integrates all variables (column (7)).

**Table 2:** Regression results of the impact of IIequality and SC on MH

**Variable**		(1)	(2)	(3)	(4)	(5)	(6)	(7)
		MH						
**IIequlity**	**KI**	−0.962*** (− 14.55)						−0.964*** (−14.19)
**SC**	**Trust**		0.150*** (− 13.590)					0.146*** (− 13.130)
	**Fair**			0.0977*** (−10.820)				0.104*** (−11.540)
	**Support**				0.002 (−0.720)			−0.005 (−1.39)
	**Relations**					0.028*** (−4.120)		0.025*** (− 3.640)
	**Jiegousc**						0.029*** (− 5.250)	0.015*** (− 2.580)
**IVC**	**Urban**	0.0809*** (−2.810)	0.169*** (−6.010)	0.187*** (−6.650)	0.183*** (− 6.480)	0.170*** (− 6.050)	0.169*** (− 6.010)	0.113*** (− 3.910)
	**Gender**	0.490*** (−14.080)	0.462*** (− 13.320)	0.456*** (− 13.140)	0.453*** (− 13.050)	0.463*** (− 13.340)	0.469*** (− 13.510)	0.480*** (− 13.740)
	**Age**	−0.002* (− 1.83)	− 0.003** (− 2.32)	− 0.003*** (− 3.12)	− 0.004*** (− 3.57)	− 0.003** (− 2.30)	− 0.002** (− 2.04)	−0.004*** (− 3.78)
	**Marriage**	0.261*** (− 7.080)	0.278*** (− 7.570)	0.295*** (− 8.030)	0.290*** (− 7.890)	0.278*** (− 7.580)	0.265*** (− 7.200)	0.285*** (− 7.700)
	**Education**	0.028*** (− 8.070)	0.041*** (− 12.070)	0.039*** (− 11.420)	0.0461*** (− 13.410)	0.0413*** (− 12.110)	0.041*** (− 11.870)	0.031*** (− 8.720)
**SCV**	**Employ**	0.066* (− 1.950)	0.071** (− 2.080)	0.059* (− 1.730)	0.072** (− 2.100)	0.071** (− 2.100)	0.064* (− 1.880)	0.053* (− 1.550)
	**Insurance**	0.124*** (− 2.690)	0.124*** (− 2.700)	0.117** (− 2.540)	0.127*** (− 2.760)	0.120*** (− 2.610)	0.119*** (− 2.580)	0.112** (− 2.420)
	**Family**	0.081*** (− 11.630)	0.062*** (− 9.070)	0.059*** (− 8.660)	0.063*** (− 9.240)	0.061*** (− 9.050)	0.058*** (− 8.460)	0.077*** (− 10.950)
	**Status**	0.067*** (− 5.600)	0.0642*** (− 5.380)	0.0571*** (− 4.770)	0.0626*** (− 5.240)	0.0523*** (− 4.260)	0.0639*** (− 5.360)	0.048*** (− 3.870)
**Living habit**	**Exercise**	0.0323*** (− 7.690)	0.0346*** (− 8.230)	0.0339*** (− 8.050)	0.0354*** (− 8.380)	0.0344*** (− 8.180)	0.0344*** (− 8.200)	0.0319*** (− 7.550)
	**Smoke**	− 0.0984*** (− 2.69)	− 0.0945*** (− 2.59)	−0.0823** (− 2.25)	−0.0883** (− 2.42)	−0.0955*** (− 2.62)	−0.0985*** (− 2.70)	−0.0825** (− 2.25)
	**Drink**	0.0704* (−1.810)	0.0811** (− 2.100)	0.0878** (− 2.270)	0.0807** (− 2.090)	0.0817** (− 2.110)	0.0785** (− 2.030)	0.0748* (− 1.920)
	**Break**	0.010 (− 0.360)	0.017 (− 0.650)	0.016 (− 0.610)	0.019 (− 0.720)	0.018 (− 0.660)	0.015 (− 0.550)	0.009 (− 0.350)
**ME**	**Satisfaction**	0.114*** (− 5.690)	0.109*** (− 5.480)	0.0819*** (− 4.090)	0.0973*** (− 4.880)	0.110*** (− 5.50)	0.111*** (− 5.560)	0.076*** (− 3.770)
	**Level**	0.148*** (− 7.890)	0.145*** (− 7.760)	0.120*** (− 6.410)	0.137*** (− 7.320)	0.144*** (− 7.730)	0.146*** (− 7.840)	0.116*** (− 6.130)
**Observations**		22, 751	22, 751	22, 751	22, 751	22, 751	22, 751	22, 751
**Province**		YES	YES	YES	YES	YES	YES	YES
***Prob*** **> F**		0.000	0.000	0.000	0.000	0.000	0.000	0.000
**Pseudo*R***^**2**^		0.040	0.036	0.040	0.038	0.036	0.036	0.047

*IIequality* Income inequality, *SC* Social capital, *ICV* Individual characteristic variables, *SCV* Social characteristic variables, *ME* Medical Environment, *MH* Mental health

*** *P* < 0.01, ** *P* < 0.05, * *P* < 0.1, robust t statistic reported in brackets, same below

**Table 3:** Regression results of the impact of IIequality and SC on PH

**Variable**		(1)	(2)	(3)	(4)	(5)	(6)	(7)
		PH						
**IIequlity**	**KI**	−0.280*** (− 4.08)						− 0.381*** (− 5.44)
**SC**	**Trust**		0.0471*** (− 4.200)					0.0460*** (−4.090)
	**Fair**			0.0674*** (−7.210)				0.0687*** (− 7.330)
	**Support**				−0.024*** (−6.82)			− 0.026*** (−7.26)
	**Relations**					0.009 (−1.280)		0.009 (−1.320)
	**Jiegousc**						−0.010* (− 1.77)	−0.013** (− 2.21)
**IVC**	**Urban**	0.043 (− 1.420)	0.0733** (− 2.450)	0.0748** (− 2.500)	0.0803*** (− 2.680)	0.0695** (− 2.330)	0.0695** (− 2.330)	0.054* (− 1.760)
	**Gender**	0.245*** (−6.670)	0.231*** (− 6.300)	0.236*** (− 6.430)	0.230*** (− 6.270)	0.238*** (− 6.480)	0.235*** (− 6.400)	0.230*** (− 6.250)
	**Age**	−0.0322*** (− 27.89)	− 0.0321*** (− 27.72)	−0.0327*** (− 28.22)	−0.0335*** (− 28.69)	−0.0324*** (− 28.02)	−0.0325*** (− 28.06)	−0.0335*** (− 28.49)
	**Marriage**	0.041 (− 1.050)	0.051 (− 1.330)	0.051 (− 1.330)	0.050 (− 1.310)	0.046 (− 1.200)	0.051 (− 1.330)	0.060 (− 1.550)
	**Education**	0.0186*** (−5.140)	0.0243*** (− 6.920)	0.0216*** (− 6.160)	0.0254*** (− 7.180)	0.0223*** (− 6.350)	0.0226*** (− 6.450)	0.0221*** (− 6.050)
**SCV**	**Employ**	0.238*** (− 6.830)	0.238*** (− 6.800)	0.235*** (− 6.700)	0.237*** (− 6.780)	0.239*** (− 6.820)	0.241*** (− 6.880)	0.237*** (− 6.770)
	**Insurance**	0.056** (− 0.130)	0.012** (− 0.030)	0.016* (− 0.040)	0.017** (− 0.040)	0.013** (− 0.030)	0.016* (− 0.040)	0.093** (− 0.230)
	**Family**	0.031 (− 0.640)	0.039 (− 0.790)	0.030 (− 0.610)	0.033 (− 0.680)	0.030 (− 0.620)	0.034 (− 0.700)	0.037 (− 0.760)
	**Status**	0.0353*** (−4.810)	0.0257*** (− 3.560)	0.0292*** (− 4.060)	0.0311*** (− 4.320)	0.0301*** (− 4.190)	0.0316*** (− 4.360)	0.034*** (− 4.610)
**Living habit**	**Exercise**	0.017*** (−1.390)	0.016** (− 1.280)	0.014** (− 1.120)	0.015*** (− 1.160)	0.012** (− 0.970)	0.016** (− 1.310)	0.010* (− 0.750)
	**Smoke**	−0.010** (− 2.48)	− 0.008* (− 1.96)	−0.010** (− 2.37)	−0.009** (− 2.22)	−0.010** (− 2.32)	−0.009** (− 2.26)	−0.009** (− 2.16)
	**Drink**	0.109*** (− 2.790)	0.109*** (− 2.780)	0.113*** (− 2.890)	0.115*** (− 2.940)	0.109*** (− 2.810)	0.111*** (− 2.830)	0.117*** (− 3.000)
	**Break**	0.291*** (−6.910)	0.299*** (− 7.100)	0.296*** (− 7.040)	0.293*** (− 6.970)	0.294*** (− 6.990)	0.296*** (− 7.020)	0.299*** (− 7.060)
**ME**	**Satisfaction**	0.102*** (−3.62)	0.0966*** (−3.42)	0.0998*** (− 3.53)	0.0982*** (− 3.48)	0.0996*** (− 3.53)	0.0987*** (− 3.49)	0.097*** (− 3.43)
	**Level**	0.0578*** (−2.800)	0.0556*** (− 2.700)	0.0477** (− 2.300)	0.0480** (− 2.330)	0.0567*** (− 2.750)	0.0561*** (− 2.720)	0.039* (− 1.870)
**Observations**		22, 751	22, 751	22, 751	22, 751	22, 751	22, 751	22, 751
**Province**		YES	YES	YES	YES	YES	YES	YES
***Prob*** **> F**		0.000	0.000	0.000	0.000	0.000	0.000	0.000
**Pseudo*R***^**2**^		0.055	0.055	0.055	0.055	0.054	0.054	0.057

*IIequality* Income inequality, *SC* Social capital, *ICV* Individual characteristic variable, *SCV* Social characteristic variable, *ME* Medical Environment, *PH* Physical health

*** *P* < 0.01, ** *P* < 0.05, * *P* < 0.1, robust t statistic reported in brackets

#### The impact of income inequality on physical and mental health

The data in column (1) of Tables 2 and 3 both show that the coefficient of effect of IIequality on PH and MH is significantly negative (MH: β = − 0.962, OR = 0.382; PH: β = − 0.280, OR = 0.756; *P* < 0.01). Integrating other variables (column (7)), the regression coefficient is still significantly negative (β = − 0.964, OR = 0.382 and β = − 0.381, OR = 0.758; *P* < 0.01). These results show that IIequality has a negative effect on PH and MH.

#### The impact of social capital on physical and mental health

This study revealed that cognitive SC has a positive effect on PH and MH. As shown in columns (2)–(6) of Tables 2 and 3, the coefficients of cognitive SC on PH are 0.0471 and 0.0674 (OR = 1.048; OR = 1.070, *P* < 0.01), and the coefficients on MH are 0.150 and 0.0977 (OR = 1.162; OR = 1.103, *P* < 0.01). Structural SC also shows a significantly effect on PH and MH, with the coefficients on MH being 0.002 (OR = 1.002, *P* > 0.1), 0.028 (OR = 1.028, *P* < 0.01), and 0.029 (OR = 1.030, *P* < 0.01), respectively, and the regression coefficients on PH being − 0.024 (OR = 0.976, *P* < 0.01), 0.009 (OR = 1.009, *P* > 0.1), − 0.010 (OR = 0.990, *P* < 0.01). Integrating other variables (column (7)), the significance level of the regression coefficient is still high (Cognitive SC(MH): β = 0.146 and 0.104, OR = 1.157 and 1.110, *P* < 0.01; Cognitive SC (PH): β = 0.0460 and 0.0687, OR = 1.047 and 1.071, *P* < 0.01; Structural SC (MH): β = − 0.005, 0.025 and 0.015, OR = 0.995, 1.025 and 1.015, *P* > 0.1, *P* < 0.01 and *P* < 0.01; Structural SC (PH): β = − 0.026, 0.009 and − 0.013, OR = 0.975, 1.009 and 0.987, *P* < 0.01, *P >* 0.1 and *P* < 0.01). The regression results can fully support that cognitive SC and structural SC can have effects on promoting the development of PH and MH.

#### The impact of covariates on physical and mental health

The columns (7) of Tables 2 and 3 show that household registration and gender have a significantly positive correlation with PH and MH (COA: 0.054, 0.230, OR = 1.056 and 1.258; 0.113, 0.480, OR = 1.120 and 1.616; *P* < 0.01; COA = Coefficient of action). These results indicate that there are household registration differences and gender differences in the inhibitory effect of IIequality on health. Factors such as the level of medical insurance and social status play a positive role in regulating PH and MH (COA: 0.093, 0.034, OR = 1.097 and 1.035; 0.112, 0.048, OR = 1.119 and 1.049; *P* < 0.01). Good living habits also have a positive effect on PH and MH, including not smoking, regular exercise, and lunch breaks. People should be encouraged to develop good habits. The higher the level of medical service satisfaction and diagnosis and treatment, the more beneficial to PH and MH (COA: 0.097, 0.039, OR = 0.907 and 1.040; 0.076, 0.116, OR = 1.079 and 1.123; *P* < 0.01), indicating the importance of the improvement of the medical environment and the level of diagnosis and treatment.

#### Robustness test

Above all, the goodness of fit of the ordered logistic regression equation was analyzed. The values of *Pseudo R*^*2*^ are maintained between 0.04–0.06, and the probability calculated by the Log-likelihood test is 0, which is less than the significance level of 0.05 (*Prob* > F). It shows that the selected IV has a significant influence on the DV, and the overall fit of the equation is good.

The results from various robustness tests are summarized in Table 4. Taking the change of the DV as an example, the effective coefficient of IIequality on self-rated health is − 0.558 (OR = 0.572), and the effect coefficient is negative. The coefficients of social trust, social justice, social support, interpersonal relationship, network density and other SC values are 0.080, 0.037, − 0.005, 0.032, − 0.004, respectively, which are proved to be significant (OR = 1.083, 1.037, 0.995, 1.032, 0.996). According to the results of a variety of robustness tests, IIequality will inhibit PH and MH, and SC plays an important role in PH and MH. This conclusion is stable and reliable.

**Table 4:** Summary results of robustness analysis

**Variable**		**(1)**	**(2)**	**(3)**	**(4)**	**(5)**	**(6)**	**(7)**
		**Health variable**	**MH**	**PH**	**MH**	**PH**	**MH**	**PH**
		**Change DV (Self-rated)**	**Change IV (Podder index)**		**Outlier test (Winsor method)**		**Change estimation model (OLS)**	
**IIequality**		− 0.558*** (−8.75)	−2.052*** (− 9.390)	−6.535*** (− 21.500)	−0.970*** (− 14.24)	−0.378*** (− 5.38)	− 2.104*** (− 15.47)	−0.147*** (− 5.34)
**SC**	**Trust**	0.080*** (− 7.490)	0.147*** (− 13.080)	0.042*** (− 3.660)	0.148*** (− 13.160)	0.048*** (− 4.180)	0.304*** (− 13.570)	0.017*** (− 3.770)
	**Fair**	0.037*** (− 4.090)	0.100*** (− 10.900)	0.066*** (− 6.860)	0.106*** (− 11.580)	0.070*** (− 7.370)	0.211*** (− 11.820)	0.024*** (− 6.580)
	**Support**	−0.005* (− 1.66)	0.004 (− 1.270)	−0.014*** (− 3.93)	−0.005 (− 1.43)	−0.026*** (− 7.26)	−0.017*** (− 2.59)	−0.010*** (− 7.28)
	**Relations**	0.032*** (− 4.760)	0.026*** (− 3.730)	0.013** (− 1.730)	0.025*** (− 3.640)	0.010 (− 1.350)	0.055*** (− 4.030)	0.004** (− 1.340)
	**Jiegousc**	− 0.004* (− 0.71)	0.034*** (− 6.040)	−0.010** (− 1.750)	0.0138** (− 2.410)	−0.0133** (− 2.22)	0.0281** (− 2.420)	−0.004 (− 1.57)
**CV**		Yes	Yes	Yes	Yes	Yes	Yes	Yes
**PDV**		Yes	Yes	Yes	Yes	Yes	Yes	Yes
**Observations**		22, 751	22, 750	22, 750	22, 296	22, 296	22, 751	22, 751
***Prob*** **> F**		0.000	0.000	0.000	0.000	0.000	0.000	0.000
**Pseudo R**^**2**^		0.051	0.114	0.121	0.048	0.058	0.046	0.073

*MH* Mental health, *PH* Physical health, *DV* Dependent variable, *IV* Independent Variable, *OLS* Ordinary Least Squares Regression, *IIequality* Income inequality, *SC* Social capital, *CV* Control variable, *PDV* Province dummy variablet

*** *P* < 0.01, ** *P* < 0.05, * *P* < 0.1, robust t statistic reported in brackets

### Heterogeneity validation

The first four columns of Table 5 show that IIequality has a negative effect on the PH and MH of both men and women but is more prominent in the female group. In most circumstances, SC has a positive effect on the PH and MH of both sexes, but its boosting effect on health performs better in the male group. As shown in the last four columns of Table 5, the deprivation effect of IIequality on the PH and MH of rural residents is higher than that of urban residents, but the overall gap is not significantly different. Different SC showed slightly different performance in different household registration groups. Cognitive SC had a greater positive effect on the PH and MH of rural residents, while structural SC exhibited a more obvious effect on the PH and MH of urban residents. Table 6 shows the analysis results showing the differences by region. According to China’s three major economic divisions, the region is divided into eastern, central, and western regions, and then the regional heterogeneity was regressed and calculated. The regression results show that the inhibitory effect of IIequality on the PH and MH of the population in different regions showed a decreasing trend from east to middle and west. There are also some regional differences in the effects of SC on PH and MH, but the effect was more significant on the population in the eastern region.

**Table 5:** Test results of gender and household registration heterogeneity

**Variable**		**(1)**	**(2)**	**(3)**	**(4)**	**(5)**	**(6)**	**(7)**	**(8)**
		**MH**	**PH**	**MH**	**PH**	**MH**	**PH**	**MH**	**PH**
		**Female**		**Male**		**Rural**		**Urban**	
**IIequality**		− 1.047*** (− 11.13)	− 0.387*** (− 4.06)	− 0.915*** (− 9.27)	− 0.386*** (− 3.81)	−0.957*** (− 10.16)	−0.319*** (− 3.23)	−0.930*** (− 9.27)	−0.273*** (− 4.59)
**SC**	**Trust**	0.134*** (− 8.650)	0.0269* (− 1.720)	0.158*** (− 9.790)	0.0637*** (− 3.880)	0.162*** (− 9.320)	0.0493*** (− 2.800)	0.153*** (− 9.450)	0.0488*** (− 2.940)
	**Fair**	0.102*** (− 8.180)	0.0643*** (− 5.040)	0.106*** (− 8.020)	0.0713*** (− 5.120)	0.115*** (− 9.320)	0.0902*** (− 3.950)	0.0886*** (− 6.610)	0.0899*** (− 6.390)
	**Support**	0.000 (− 0.100)	− 0.030*** (− 6.03)	− 0.0101** (− 2.06)	−0.0209*** (− 3.98)	−0.003 (− 0.68)	−0.0125** (− 2.49)	−0.004 (− 0.80)	−0.037*** (− 7.38)
	**Relations**	0.011 (− 1.180)	0.000 (− 0.010)	0.040*** (− 4.040)	0.022** (− 2.130)	0.022** (− 2.370)	0.002 (− 0.170)	0.028*** (− 2.800)	0.018* (− 1.740)
	**Jiegousc**	0.013 (− 1.630)	− 0.020** (− 2.40)	0.017** (− 2.040)	− 0.007 (− 0.81)	0.013** (− 1.510)	− 0.023** (− 2.53)	0.014* (− 1.650)	− 0.028** (− 1.18)
**CV**		Yes	Yes	Yes	Yes	Yes	Yes	Yes	Yes
**PDV**		Yes	Yes	Yes	Yes	Yes	Yes	Yes	Yes
**Observations**		11,879	11,879	10,872	10,872	11,546	11,546	11,205	11,205
***Prob*** **> F**		0.000	0.000	0.000	0.000	0.000	0.000	0.000	0.000
**Pseudo R**^**2**^		0.042	0.058	0.046	0.051	0.049	0.062	0.042	0.056

*MH* Mental health, *PH* Physical health, *IIequality* Income inequality, *SC* Social capital, *CV* Control variable, *PDV* Province dummy variablet

*** *P* < 0.01, ** *P* < 0.05, * *P* < 0.1, robust t statistic reported in brackets

**Table 6:** Results of regional heterogeneity test

**Variable**		**(1)**	**(2)**	**(3)**	**(4)**	**(5)**	**(6)**
		**MH**	**PH**	**MH**	**PH**	**MH**	**PH**
		**Eastern region**		**Central region**		**Western region**	
**IIequality**		−1.104*** (− 11.27)	−0.529*** (− 5.24)	−1.088*** (− 7.12)	−0.352** (− 2.24)	−0.693*** (− 5.71)	− 0.207* (− 1.64)
**SC**	**Trust**	0.148*** (−9.280)	0.047*** (− 2.900)	0.123*** (− 4.870)	0.038 (− 1.460)	0.147*** (− 7.410)	0.0359* (− 1.780)
	**Fair**	0.125*** (− 7.950)	0.093*** (− 6.730)	0.115*** (− 5.740)	0.055*** (− 2.680)	0.097*** (− 6.000)	0.042** (− 2.550)
	**Support**	− 0.011** (− 2.23)	− 0.028*** (− 5.51)	−0.002 (− 0.22)	−0.030*** (− 3.88)	0.005 (− 0.760)	−0.017*** (− 2.63)
	**Relations**	0.048*** (− 4.750)	0.001 (− 0.130)	0.024* (− 1.640)	0.014 (− 0.850)	− 0.007 (− 0.60)	0.017 (− 1.440
	**Jiegousc**	0.0123* (− 1.650)	− 0.008* (− 1.08)	0.002* (− 0.130)	− 0.036** (− 2.45)	0.026** (− 2.180)	−0.015 (− 1.26)
**CV**		Yes	Yes	Yes	Yes	Yes	Yes
**PDV**		Yes	Yes	Yes	Yes	Yes	Yes
**Observations**		10, 952	10, 952	4, 926	4, 926	6, 873	6, 873
***Prob*** **> F**		0.000	0.000	0.000	0.000	0.000	0.000
**Pseudo*R***^**2**^		0.046	0.054	0.037	0.059	0.043	0.060

*MH* Mental health, *PH* Physical health, *IIequality* Income inequality, *SC* Social capital, *CV* Control variable, *PDV* Province dummy variablet

*** *P* < 0.01, ** *P* < 0.05, * *P* < 0.1, robust t statistic reported in brackets

### Intermediary effect validation

The mediation effect in this study was designed to analyze the data (*N* = 22,751) by the KHB method. As shown in Table 7, it can be observed that there is a partial mediating effect of social capital collection in the path of IIequality on PH and MH. After controlling for confounding variables such as CVs and PDV, the mediating effect of cognitive SC in the total effect of income inequality on physical and mental health accounted for 6.95, 4.59, 3.44, 2.74% (OR = 0.919, 0.055, 0.977, 0.981; *P* < 0.001). As for the three structural SCs, the IE of Network density on the association of IIequality with MH was 2.98% (OR = 0.965, *P* < 0.001), at the same time, the IE of Social support on the association of IIequality with PH was 13.22% (OR = 1.095, *P* < 0.001).

**Table 7:** Test results of the mediation effect of social capital

**DV**	**IV**		**Coef.**	**OR**	**Std. Err.**	**z**	***P* > |z|**	**Mediation percentage**
**MH**	**Sense of Social trust**	TE	−1.211	0.298	0.052	−23.330	0.000	6.95%
		DE	−1.127	0.324	0.052	−21.670	0.000	
		IE	−0.084	0.919	0.008	−10.230	0.000	
	**Sense of Social fairness**	TE	−1.205	0.300	0.052	−23.300	0.000	4.59%
		DE	−1.260	0.284	0.052	−24.170	0.000	
		IE	0.055	1.057	0.007	8.220	0.000	
	**Social support**	TE	−1.201	0.301	0.052	−23.240	0.000	1.21%
		DE	−1.216	0.297	0.053	−23.150	0.000	
		IE	0.015	1.015	0.009	1.570	0.117	
	**Personal relationship**	TE	−1.202	0.301	0.052	−23.240	0.000	0.10%
		DE	−1.200	0.301	0.052	−23.220	0.000	
		IE	−0.001	0.999	0.002	−0.630	0.526	
	**Network density**	TE	−1.202	0.301	0.052	−23.260	0.000	2.98%
		DE	−1.166	0.312	0.052	−22.280	0.000	
		IE	−0.036	0.965	0.009	−4.080	0.000	
**PH**	**Sense of Social trust**	TE	−0.688	0.503	0.053	−12.990	0.000	3.44%
		DE	−0.664	0.515	0.053	−12.510	0.000	
		IE	−0.024	0.977	0.005	−5.190	0.000	
	**Sense of Social fairness**	TE	−0.687	0.503	0.053	−12.980	0.000	2.74%
		DE	−0.668	0.513	0.053	−12.550	0.000	
		IE	−0.019	0.981	0.006	−3.060	0.002	
	**Social support**	TE	−0.687	0.503	0.053	−12.990	0.000	13.22%
		DE	−0.778	0.459	0.053	−12.510	0.000	
		IE	0.091	1.095	0.005	−5.190	0.000	
	**Personal relationship**	TE	−0.687	0.503	0.053	−12.980	0.000	0.01%
		DE	−0.687	0.503	0.053	−12.980	0.000	
		IE	0.000	1.000	0.000	−0.360	0.720	
	**Network density**	TE	−0.687	0.503	0.053	−12.980	0.000	1.00%
		DE	−0.680	0.507	0.054	−12.680	0.000	
		IE	−0.007	0.993	0.009	−0.770	0.443	

Mediation percentage is calculated by IE/TE × 100

*DV* Dependent variable, *IV* Independent Variable, *MH* Mental health, *PH* Physical health, *TE* Total effect, *DE* Direct effect, *IE* Intermediary effect

## Discussion

Although it is not uncommon to apply the KI to the measurement of IIequality [40, 41], this study extended it to the collaborative study of IIequality, SC, and PH and MH for the first time. Especially, this paper has applied this index to data from a developing country milieu through more sophisticated regression models.

The outcome of the research provides important support to demonstrate the negative impact of IIequality on PH and MH. After controlling the individual characteristics, family characteristics, medical service quality, and SC variables that affect PH and MH, we note that IIequality will directly affect PH and MH. However, the results reveal that the negative effect of IIequality on the mental health of an individual is more obvious than the negative effects on physical health. Consistent with the extant works, the conclusions of this study support the hypothesis that as IIequality increases, the individual’s physical and mental health will decline. From the theoretical point of view, IIequality will deprive low-income people of their ability to obtain medical resources which leads to a diminution of the quality of the living environment which can directly affect their health [42]. At the same time, IIequality stimulates a serious social and psychological shadow, which can cause anxiety and stress and ultimately damage MH [10]. The empirical results are consistent with the relative income hypothesis and income deprivation hypothesis. With the development of complex social situations such as economic globalization, miniaturization of family populations, and aging population aging, more attention needs to be paid to the adverse consequences caused by IIequality [43] and improvement measures. This study has demonstrated the positive effect of SC on PH and MH and shown that the coefficients of the five SC indicators are positive in most circumstances, suggesting the correlation of SC with the PH and MH. A novelty in this study is that when SC was decomposed into two identifiable forms, the study affirmed the fact that shows that both cognitive SC and structural SC can influence PH and MH, which is consistent with the research conclusions of other scholars [44]. As an informal system, SC can guide more social participation and social activities, and promote the development of MH and healthy behaviors [45, 46].

IIequality at the individual level can reflect the relative degree of deprivation of income. From the comparison of the coefficient values of the individual IIequality effect and the IIequality effect of other variables of cointegration, it is revealed that the negative effect of the cointegration coefficient is more obvious (− 0.964 < − 0.962; − 0.381 < − 0.280; OR = 0.382, 0.382, 0.758, 0.756; *P* < 0.01). The results of this study strengthen the explanatory effect of relative deprivation on the theory of IIequality. Relative deprivation can affect PH and MH through material pathways, psychological pathways, and acting on SC [5]. The path of IIequality on PH and MH may involve the interaction of multiple intermediary variables, which may result in differences in the COA due to intermediary confusion and ranking issues [47]. A coherent study of IIequality, SC and PH and MH is needed to further clarify the mediating role of SC in the path of IIequality on PH and MH. Our results show that both cognitive SC (social justice and social trust) and structural SC (social support and network density) have IEs. It is necessary to guard against the erosion of SC caused by IIequality, which may result in a double cumulative effect of income disadvantage and social resource disadvantage [48].

It is interesting to note that women’s PH and MH are more vulnerable to the negative effects of IIequality relative to that of the men, but the positive effects of SC are not obvious. The cause of this observation may be due to differences in physiology and social status. In terms of education, work, social resources and other aspects, women’ opportunities are more unequal than men’s, resulting in women’s income disadvantage and lack of social resources [49]. The same problem also appears between rural and urban residents. The deprivation effect of IIequality on the PH and MH of rural residents is higher than that of urban residents, but the effect of rural residents’ structural SC on health promotion is still at a disadvantage. This may be due to China’s unique urban-rural dual structure. Because of the nature of the institutional division of urban and rural areas, there are differences in wealth accumulation and income channels among urban and rural residents [50]. And the institutional dividend, family and social resources of urban residents are still significantly better than those in rural areas [51]. More attention needs to be paid to the heterogeneity of the impact of different influencing factors on PH and MH and take targeted measures for different groups of people. This is where follow-up research needs to be deepened.

This study used China’s 2018 CFPS, a typical representative micro-database for empirical analysis. The data sample size is large (*N* = 22,751) and the survey content focuses on many aspects such as society, income, people’s livelihood, education, health, etc., all of which are first-hand data, ensuring the applicability of the research data. After multiple rounds of problem correction, these data can truly reflect the PH and MH of Chinese people and related influencing factors, laying a foundation for the reliability of research conclusions. The research comprehensively adopts the robustness test method to repeatedly validate the research conclusions, including methods such as replacing IV or DV, changing the estimation model, and winsor’s tailing treatment method. The research conclusions are consistent, indicating the stability and reliability of the research conclusions. Finally, The KHB analysis results prove that after controlling for variables such as individual characteristics, social characteristics, and living habits, SC does have a partial mediating role in the path of IIequality on PH and MH [44]. In addition, the study did not only analyze the impact of overall sample data on PH and MH, but also conducted partial analysis by gender, household registration, and population, and discusses the differences in factors affecting PH and MH of different populations, which is an expansion from the whole to the part.

Research findings might provide some enlightenment for developing countries to improve national health. At first, we should attach great importance to the weakening effect of inequality on physical and mental health, pay attention to income equity, be alert to the vicious circle of health-poverty traps, and take active interventions to decouple income from health [2, 3]; Secondly, take targeted measures for the physical and mental health of groups with special difficulties. Female population [49], rural population and poor population in underdeveloped areas are the focus of attention [50, 51]. For this group of people, there is a need to strengthen the construction of a preferential social security system, increase educational opportunities, and pay attention to employment fairness; Finally, it is important to actively play an intermediary role of social capital, inequality buffer negative effects of physical and mental health. The development of SC, especially cognitive SC, can enhance people’s perception of social fairness and trust, reduce resistance to economic development, enhance transaction transparency, provide active social supervision and management, and improve the efficiency of economic operations. Individuals should also attach importance to the construction and development of their social networks. Social networks can not only broaden the source of resource information, but also have the function of insurance apportionment, enhance the individual’s ability to resist risks, provide individuals with emotional support and material support, and rely on social support and resources. Mutual assistance gets rid of the disadvantages of its development [18–20].

## Limitation

This research also has a few limitations. Firstly, the data in this study are cross-sectional data. The conclusion can only explain the correlation between variables, and the direction of the influence path cannot be determined. There may be problems such as missing variables. At the same time, the reverse causality problem cannot be ruled out [52]. The impact of IIequality on SC and PH and MH may have a time lag [53]. Studies have shown that there is a considerable lag time for the impact of IIequality on health, and current IIequality may have an important impact on later health [54]. This study did not include the influence of time frame on the research conclusions, which may limit the interpretation of the regression results. At the same time, the selected analysis indicators have a certain degree of subjectivity. For example, SC variables such as social justice and social trust are mostly subjective variables, and there are potential psychological characteristics to confuse the relationship between independent variables and dependent variables [55].

## Conclusions

This study shows that IIequality can endanger PH and MH, and SC is beneficial to PH and MH, and there is a mediating effect in the path of IIequality on PH and MH. Our new findings demonstrate that there are gender differences, urban-rural differences, and regional differences in the effects of IIequality and SC on PH and MH. These findings can be used as new evidence that IIequality is harmful to PH and MH. This study also demonstrates from the two dimensions of cognition and structure that IIequality can affect PH and MH through the effect of various types of SC and provide a way for developing countries to improve their national PH and MH. Enlightenment. Future research should further examine the time lag of the impact of IIequality on SC and PH and MH.

## Supplementary Information

**Additional file 1: Table S1.** Multicollinearity Test Results. **Table S2.** Regression results of the impact of IIequality and SC on MH. **Table S3.** Regression results of the impact of IIequality and SC on PH. **Table S4.** Summary results of robustness analysis. **Table S5.** Test results of gender and household registration heterogeneity. **Table S6.** Results of regional heterogeneity test.

## Data Availability

The data that support the findings of this study are available from the Institute of Social of Peking University (ISSS). Data and any supplementary material related to this article can be obtained from the correspondence author on request.

## Abbreviations

CFPS: China Family Panel Studies Database
COA: Coefficient of action
PDV: Province dummy variable
CV: Control variable
DV: Dependent variable
IV: Independent variable
ICV: Individual characteristic variables
SCV: Social characteristic variables
KI: Kakwani index
MH: Mental health
PH: Physical health
SC: Social capital
IIequality: Income inequality
ME: Medical environment
OR: Odds Ratio
TE: Total effect
DE: Direct effect
IE: Intermediary effect
