# Supplementary material for: An empirical analysis of the impact of income inequality and social capital on physical and mental health - take China’s micro-database analysis as an example
Source: Int J Equity Health. 2021 Nov 6;20:241. doi: 10.1186/s12939-021-01560-w (PMC8571851; doi:10.1186/s12939-021-01560-w)
Supplement: Supplementary file 1 — Additional file 1: Table S1. Multicollinearity Test Results. Table S2. Regression results of the impact of IIequality and SC on MH. Table S3. Regression results of the impact of IIequality and SC on PH. Table S4. Summary results of robustness analysis. Table S5. Test results of gender and household registration heterogeneity. Table S6. Results of regional heterogeneity test. [file 12939_2021_1560_MOESM1_ESM.docx]

**Additional file 1**

**Table S1. Multicollinearity Test Results**

| **Variable** | ***β*** | **Beta** | **t** | ***P*** | **TOL** | **VIF** |
| --- | --- | --- | --- | --- | --- | --- |
| Cons | 20.283 |  | 75.738 | .000 |  |  |
| Kakwani | -2.225 | -.136 | -17.876 | .000 | .692 | 1.444 |
| support | -.019 | -.019 | -2.871 | .004 | .954 | 1.048 |
| trust | .360 | .120 | 18.269 | .000 | .928 | 1.077 |
| fair | .205 | .078 | 11.799 | .000 | .908 | 1.102 |
| relation | .057 | .028 | 4.279 | .000 | .936 | 1.069 |
| jiegousc | .037 | .022 | 3.362 | .001 | .930 | 1.076 |
| urban | .251 | .031 | 4.411 | .000 | .803 | 1.245 |
| gender | .964 | .119 | 14.165 | .000 | .562 | 1.778 |
| age | -.009 | -.036 | -4.260 | .000 | .573 | 1.745 |
| marriage | .703 | .066 | 9.805 | .000 | .877 | 1.141 |
| edu | .078 | .095 | 11.307 | .000 | .569 | 1.757 |
| employ | .115 | .012 | 1.729 | .084 | .840 | 1.190 |
| familynumber | .144 | .074 | 10.894 | .000 | .857 | 1.167 |
| status | .123 | .033 | 5.076 | .000 | .928 | 1.077 |
| exercise | .072 | .059 | 8.953 | .000 | .933 | 1.072 |
| smoke | -.224 | -.025 | -3.145 | .002 | .612 | 1.633 |
| drink | .269 | .024 | 3.539 | .000 | .855 | 1.170 |
| break | .026 | .003 | .505 | .613 | .964 | 1.037 |
| zlSatisfaction | .149 | .030 | 3.834 | .000 | .648 | 1.543 |
| zllevel | .227 | .050 | 6.334 | .000 | .648 | 1.543 |

**Table S2.** **Regression results of the impact of IIequality and SC on MH**

| **Variable** | | (1) | (2) | (3) | (4) | (5) | (6) | (7) |
| --- | --- | --- | --- | --- | --- | --- | --- | --- |
|  |  | MH | | | | | | |
| **IIequlity** | **KI** | -0.962*** |  |  |  |  |  | -0.964*** |
|  |  | (-14.55) |  |  |  |  |  | (-14.19) |
|  |  | [0.382] |  |  |  |  |  | [0.382] |
| **SC** | **Trust** |  | 0.150*** |  |  |  |  | 0.146*** |
|  |  |  | (-13.590) |  |  |  |  | (-13.130) |
|  |  |  | [1.162] |  |  |  |  | [1.157] |
|  | **Fair** |  |  | 0.0977*** |  |  |  | 0.104*** |
|  |  |  |  | (-10.820) |  |  |  | (-11.540) |
|  |  |  |  | [1.103] |  |  |  | [1.110] |
|  | **Support** |  |  |  | 0.002 |  |  | -0.005 |
|  |  |  |  |  | (-0.720) |  |  | (-1.39) |
|  |  |  |  |  | [1.002] |  |  | [0.995] |
|  | **Relations** |  |  |  |  | 0.028*** |  | 0.025*** |
|  |  |  |  |  |  | (-4.120) |  | (-3.640) |
|  |  |  |  |  |  | [1.028] |  | [1.025] |
|  | **Jiegousc** |  |  |  |  |  | 0.029*** | 0.015*** |
|  |  |  |  |  |  |  | (-5.250) | (-2.580) |
|  |  |  |  |  |  |  | [1.030] | [1.015] |
| **IVC** | **Urban** | 0.0809*** | 0.169*** | 0.187*** | 0.183*** | 0.170*** | 0.169*** | 0.113*** |
|  |  | (-2.810) | (-6.010) | (-6.650) | (-6.480) | (-6.050) | (-6.010) | (-3.910) |
|  |  |  |  |  |  |  |  | [1.120] |
|  | **Gender** | 0.490*** | 0.462*** | 0.456*** | 0.453*** | 0.463*** | 0.469*** | 0.480*** |
|  |  | (-14.080) | (-13.320) | (-13.140) | (-13.050) | (-13.340) | (-13.510) | (-13.740) |
|  |  |  |  |  |  |  |  | [1.616] |
|  | **Age** | -0.00204* | -0.00259** | -0.00347*** | -0.00401*** | -0.00256** | -0.00228** | -0.00428*** |
|  |  | (-1.83) | (-2.32) | (-3.12) | (-3.57) | (-2.30) | (-2.04) | (-3.78) |
|  |  |  |  |  |  |  |  | [0.996] |
|  | **Marriage** | 0.261*** | 0.278*** | 0.295*** | 0.290*** | 0.278*** | 0.265*** | 0.285*** |
|  |  | (-7.080) | (-7.570) | (-8.030) | (-7.890) | (-7.580) | (-7.200) | (-7.700) |
|  |  |  |  |  |  |  |  | [1.330] |
|  | **Edu** | 0.0284*** | 0.0412*** | 0.0390*** | 0.0461*** | 0.0413*** | 0.0405*** | 0.0310*** |
|  |  | (-8.070) | (-12.070) | (-11.420) | (-13.410) | (-12.110) | (-11.870) | (-8.720) |
|  |  |  |  |  |  |  |  | [1.032] |
| **SVC** | **Employ** | 0.0662* | 0.0706** | 0.0588* | 0.0716** | 0.0714** | 0.0640* | 0.053* |
|  |  | (-1.950) | (-2.080) | (-1.730) | (-2.100) | (-2.100) | (-1.880) | (-1.550) |
|  |  |  |  |  |  |  |  | [1.054] |
|  | **Insurance** | 0.124*** | 0.124*** | 0.117** | 0.127*** | 0.120*** | 0.119*** | 0.112** |
|  |  | (-2.690) | (-2.700) | (-2.540) | (-2.760) | (-2.610) | (-2.580) | (-2.420) |
|  |  |  |  |  |  |  |  | [1.119] |
|  | **Family** | 0.0812*** | 0.0618*** | 0.0587*** | 0.0628*** | 0.0614*** | 0.0575*** | 0.0773*** |
|  |  | (-11.630) | (-9.070) | (-8.660) | (-9.240) | (-9.050) | (-8.460) | (-10.950) |
|  |  |  |  |  |  |  |  | [1.080] |
|  | **Status** | 0.0669*** | 0.0642*** | 0.0571*** | 0.0626*** | 0.0523*** | 0.0639*** | 0.048*** |
|  |  | (-5.600) | (-5.380) | (-4.770) | (-5.240) | (-4.260) | (-5.360) | (-3.870) |
|  |  |  |  |  |  |  |  | [1.049] |
| **Living habit** | **Exercise** | 0.0323*** | 0.0346*** | 0.0339*** | 0.0354*** | 0.0344*** | 0.0344*** | 0.0319*** |
|  |  | (-7.690) | (-8.230) | (-8.050) | (-8.380) | (-8.180) | (-8.200) | (-7.550) |
|  |  |  |  |  |  |  |  | [1.032] |
|  | **Smoke** | -0.0984*** | -0.0945*** | -0.0823** | -0.0883** | -0.0955*** | -0.0985*** | -0.0825** |
|  |  | (-2.69) | (-2.59) | (-2.25) | (-2.42) | (-2.62) | (-2.70) | (-2.25) |
|  |  |  |  |  |  |  |  | [0.921] |
|  | **Drink** | 0.0704* | 0.0811** | 0.0878** | 0.0807** | 0.0817** | 0.0785** | 0.0748* |
|  |  | (-1.810) | (-2.100) | (-2.270) | (-2.090) | (-2.110) | (-2.030) | (-1.920) |
|  |  |  |  |  |  |  |  | [1.078] |
|  | **Break** | 0.010 | 0.017 | 0.016 | 0.019 | 0.018 | 0.015 | 0.009 |
|  |  | (-0.360) | (-0.650) | (-0.610) | (-0.720) | (-0.660) | (-0.550) | (-0.350) |
|  |  |  |  |  |  |  |  | [1.009] |
| **ME** | **Satisfaction** | 0.114*** | 0.109*** | 0.0819*** | 0.0973*** | 0.110*** | 0.111*** | 0.076*** |
|  |  | (-5.690) | (-5.480) | (-4.090) | (-4.880) | (-5.50) | (-5.560) | (-3.770) |
|  |  |  |  |  |  |  |  | [1.079] |
|  | **Level** | 0.148*** | 0.145*** | 0.120*** | 0.137*** | 0.144*** | 0.146*** | 0.116*** |
|  |  | (-7.890) | (-7.760) | (-6.410) | (-7.320) | (-7.730) | (-7.840) | (-6.130) |
|  |  |  |  |  |  |  |  | [1.123] |
| **Observations** | | 22751 | 22751 | 22751 | 22751 | 22751 | 22751 | 22751 |
| **Province** | | YES | YES | YES | YES | YES | YES | YES |
| **Prob>F** | | 0.000 | 0.000 | 0.000 | 0.000 | 0.000 | 0.000 | 0.000 |
| **pseudo R2** | | 0.040 | 0.036 | 0.040 | 0.038 | 0.036 | 0.036 | 0.047 |

***Note****:* *** *P* < 0.01, ** *P* < 0.05, * *P* < 0.1, Z value reported in brackets, OR value reported in square brackets, same below.

OR = Odds Ratio. IIequality = Income inequality, SC = Social capital, ICV = Individual characteristic variables, SCV = Social characteristic variables, ME = Medical Environment, MH = Mental health.

**Table S3. Regression results of the impact of IIequality and SC on PH**

| **Variable** | | (1) | (2) | (3) | (4) | (5) | (6) | (7) |
| --- | --- | --- | --- | --- | --- | --- | --- | --- |
|  |  | PH | | | | | | |
| **IIequlity** | **KI** | -0.280*** |  |  |  |  |  | -0.381*** |
|  |  | (-4.08) |  |  |  |  |  | (-5.44) |
|  |  | [0.756] |  |  |  |  |  | [0.758] |
| **SC** | **Trust** |  | 0.0471*** |  |  |  |  | 0.0460*** |
|  |  |  | (-4.200) |  |  |  |  | (-4.090) |
|  |  |  | [1.048] |  |  |  |  | [1.047] |
|  | **Fair** |  |  | 0.0674*** |  |  |  | 0.0687*** |
|  |  |  |  | (-7.210) |  |  |  | (-7.330) |
|  |  |  |  | [1.070] |  |  |  | [1.071] |
|  | **Support** |  |  |  | 0.024*** |  |  | 0.026*** |
|  |  |  |  |  | (-6.82) |  |  | (-7.26) |
|  |  |  |  |  | [0.976] |  |  | [0.975] |
|  | **Relations** |  |  |  |  | 0.009 |  | 0.009 |
|  |  |  |  |  |  | (-1.280) |  | (-1.320) |
|  |  |  |  |  |  | [1.009] |  | [1.009] |
|  | **Jiegousc** |  |  |  |  |  | 0.010* | 0.013** |
|  |  |  |  |  |  |  | (-1.77) | (-2.21) |
|  |  |  |  |  |  |  | [0.990] | [0.987] |
| **IVC** | **Urban** | 0.043 | 0.0733** | 0.0748** | 0.0803*** | 0.0695** | 0.0695** | 0.054* |
|  |  | (-1.420) | (-2.450) | (-2.500) | (-2.680) | (-2.330) | (-2.330) | (-1.760) |
|  |  |  |  |  |  |  |  | [1.056] |
|  | **Gender** | 0.245*** | 0.231*** | 0.236*** | 0.230*** | 0.238*** | 0.235*** | 0.230*** |
|  |  | (-6.670) | (-6.300) | (-6.430) | (-6.270) | (-6.480) | (-6.400) | (-6.250) |
|  |  |  |  |  |  |  |  | [1.258] |
|  | **Age** | -0.0322*** | -0.0321*** | -0.0327*** | -0.0335*** | -0.0324*** | -0.0325*** | -0.0335*** |
|  |  | (-27.89) | (-27.72) | (-28.22) | (-28.69) | (-28.02) | (-28.06) | (-28.49) |
|  |  |  |  |  |  |  |  | [0.967] |
|  | **Marriage** | 0.041 | 0.051 | 0.051 | 0.050 | 0.046 | 0.051 | 0.060 |
|  |  | (-1.050) | (-1.330) | (-1.330) | (-1.310) | (-1.200) | (-1.330) | (-1.550) |
|  |  |  |  |  |  |  |  | [1.062] |
|  | **Edu** | 0.0186*** | 0.0243*** | 0.0216*** | 0.0254*** | 0.0223*** | 0.0226*** | 0.0221*** |
|  |  | (-5.140) | (-6.920) | (-6.160) | (-7.180) | (-6.350) | (-6.450) | (-6.050) |
|  |  |  |  |  |  |  |  | [1.022] |
| **SVC** | **Employ** | 0.238*** | 0.238*** | 0.235*** | 0.237*** | 0.239*** | 0.241*** | 0.237*** |
|  |  | (-6.830) | (-6.800) | (-6.700) | (-6.780) | (-6.820) | (-6.880) | (-6.770) |
|  |  |  |  |  |  |  |  | [1.267] |
|  | **Insurance** | 0.056** | 0.012** | 0.016* | 0.017** | 0.013** | 0.016* | 0.093** |
|  |  | (-0.130) | (-0.030) | (-0.040) | (-0.040) | (-0.030) | (-0.040) | (-0.230) |
|  |  |  |  |  |  |  |  | [1.097] |
|  | **Family** | 0.031 | 0.039 | 0.030 | 0.033 | 0.030 | 0.034 | 0.037 |
|  |  | (-0.640) | (-0.790) | (-0.610) | (-0.680) | (-0.620) | (-0.700) | (-0.760) |
|  |  |  |  |  |  |  |  | [1.038] |
|  | **Status** | 0.0353*** | 0.0257*** | 0.0292*** | 0.0311*** | 0.0301*** | 0.0316*** | 0.034*** |
|  |  | (-4.810) | (-3.560) | (-4.060) | (-4.320) | (-4.190) | (-4.360) | (-4.610) |
|  |  |  |  |  |  |  |  | [1.035] |
| **Living habit** | **Exercise** | 0.017*** | 0.016** | 0.014** | 0.015*** | 0.012** | 0.016** | 0.010* |
|  |  | (-1.390) | (-1.280) | (-1.120) | (-1.160) | (-0.970) | (-1.310) | (-0.750) |
|  |  |  |  |  |  |  |  | [1.010] |
|  | **Smoke** | -0.0103** | -0.00818* | -0.00988** | -0.00924** | -0.00968** | -0.00939** | -0.00905** |
|  |  | (-2.48) | (-1.96) | (-2.37) | (-2.22) | (-2.32) | (-2.26) | (-2.16) |
|  |  |  |  |  |  |  |  | [0.991] |
|  | **Drink** | 0.109*** | 0.109*** | 0.113*** | 0.115*** | 0.109*** | 0.111*** | 0.117*** |
|  |  | (-2.790) | (-2.780) | (-2.890) | (-2.940) | (-2.810) | (-2.830) | (-3.000) |
|  |  |  |  |  |  |  |  | [1.124] |
|  | **Break** | 0.291*** | 0.299*** | 0.296*** | 0.293*** | 0.294*** | 0.296*** | 0.299*** |
|  |  | (-6.910) | (-7.100) | (-7.040) | (-6.970) | (-6.990) | (-7.020) | (-7.060) |
|  |  |  |  |  |  |  |  | [1.348] |
| **ME** | **Satisfaction** | 0.102*** | 0.0966*** | 0.0998*** | 0.0982*** | 0.0996*** | 0.0987*** | 0.097*** |
|  |  | (-3.62) | (-3.42) | (-3.53) | (-3.48) | (-3.53) | (-3.49) | (-3.43) |
|  |  |  |  |  |  |  |  | [0.907] |
|  | **Level** | 0.0578*** | 0.0556*** | 0.0477** | 0.0480** | 0.0567*** | 0.0561*** | 0.039* |
|  |  | (-2.800) | (-2.700) | (-2.300) | (-2.330) | (-2.750) | (-2.720) | (-1.870) |
|  |  |  |  |  |  |  |  | [1.040] |
| **Observations** | | 22751 | 22751 | 22751 | 22751 | 22751 | 22751 | 22751 |
| **Province** | | YES | YES | YES | YES | YES | YES | YES |
| **Prob>F** | | 0.000 | 0.000 | 0.000 | 0.000 | 0.000 | 0.000 | 0.000 |
| **pseudo R2** | | 0.055 | 0.055 | 0.055 | 0.055 | 0.054 | 0.054 | 0.057 |

***Note****:* *** *P* < 0.01, ** *P* < 0.05, * *P* < 0.1, Z value reported in brackets, OR value reported in square brackets, same below.

OR = Odds Ratio. IIequality = Income inequality, SC = Social capital, ICV = Individual characteristic variables, SCV = Social characteristic variables, ME = Medical Environment, PH = Physical health.

**Table S4. Summary results of robustness analysis**

| **Variable** | | **(1)** | **(2)** | **(3)** | **(4)** | **(5)** | **(6)** | **(7)** |
| --- | --- | --- | --- | --- | --- | --- | --- | --- |
|  |  | **Health variable** | **MH** | **PH** | **MH** | **PH** | **MH** | **PH** |
|  | | **Change DV**  **（Self-rated）** | **Change IV**  **（Podder index）** | | **Outlier test**  **（Winsor method）** | | **Change estimation model**  **（OLS）** | |
| **IIequality** | | -0.558*** | -2.052*** | -6.535*** | -0.970*** | -0.378*** | -2.104*** | -0.147*** |
|  |  | (-8.75) | (-9.390) | (-21.500) | (-14.24) | (-5.38) | (-15.47) | (-5.34) |
|  |  | [0.572] | [7.786] | [8.951] | [0.379] | [0.685] |  |  |
| **SC** | **Trust** | 0.080*** | 0.147*** | 0.042*** | 0.148*** | 0.048*** | 0.304*** | 0.017*** |
|  |  | (-7.490) | (-13.080) | (-3.660) | (-13.160) | (-4.180) | (-13.570) | (-3.770) |
|  |  | [1.083] | [1.158] | [1.043] | [1.160] | [1.049] |  |  |
|  | **Fair** | 0.037*** | 0.100*** | 0.066*** | 0.106*** | 0.070*** | 0.211*** | 0.024*** |
|  |  | (-4.090) | (-10.900) | (-6.860) | (-11.580) | (-7.370) | (-11.820) | (-6.580) |
|  |  | [1.037] | [1.105] | [1.068] | [1.112] | [1.073] |  |  |
|  | **Support** | -0.005* | 0.004 | -0.014*** | -0.005 | -0.026*** | -0.017*** | -0.010*** |
|  |  | (-1.66) | (-1.270) | (-3.93) | (-1.43) | (-7.26) | (-2.59) | (-7.28) |
|  |  | [0.995] | [1.004] | [0.986] | [0.995] | [0.975] |  |  |
|  | **Relations** | 0.032*** | 0.026*** | 0.013** | 0.025*** | 0.010 | 0.055*** | 0.004** |
|  |  | (-4.760) | (-3.730 | (-1.730) | (-3.640) | (-1.350) | (-4.030) | (-1.340 |
|  |  | [1.032] | [1.026] | [1.013] | [1.025] | [1.010] |  |  |
|  | **Jiegousc** | -0.004* | 0.034*** | -0.010** | 0.0138** | -0.0133** | 0.0281** | -0.004 |
|  |  | (-0.71) | (-6.040) | (-1.750) | (-2.410) | (-2.22) | (-2.420) | (-1.57) |
|  |  | [0.996] | [1.035] | [1.010] | [1.014] | [0.987] |  |  |
| **CV** | | Yes | Yes | Yes | Yes | Yes | Yes | Yes |
| **PDV** | | Yes | Yes | Yes | Yes | Yes | Yes | Yes |
| **Observations** | | 22，751 | 22，750 | 22，750 | 22，296 | 22，296 | 22751 | 22751 |
| **Prob>F** | | 0.000 | 0.000 | 0.000 | 0.000 | 0.000 | 0.000 | 0.000 |
| **Pseudo R^2^** | | 0.051 | 0.114 | 0.121 | 0.048 | 0.058 | 0.046 | 0.073 |

***Note***: Only some or values are reported in the text. OLS regression has no or, so it is not reported.

MH = Mental health, PH = Physical health, DV = Dependent variable, IV = Independent Variable, OLS = Ordinary Least Squares Regression, IIequality = Income inequality, SC = Social capital, CV = Control variable, PDV = Province dummy variablet.

**Table S5.** Test results of gender and household registration heterogeneity

| **Variable** | | **(1)** | **(2)** | **(3)** | **(4)** | **(5)** | **(6)** | **(7)** | **(8)** |
| --- | --- | --- | --- | --- | --- | --- | --- | --- | --- |
|  | | **MH** | **PH** | **MH** | **PH** | **MH** | **PH** | **MH** | **PH** |
|  | | **Female** | | **Male** | | **Rural** | | **Urban** | |
| **IIequality** | | -1.047*** | -0.387*** | -0.915*** | -0.386*** | -0.957*** | -0.319*** | -0.930*** | -0.273*** |
|  |  | (-11.13) | (-4.06) | (-9.27) | (-3.81) | (-10.16) | (-3.23) | (-9.27) | (-4.59) |
|  |  | [0.351] | [0.679] | [0.401] | [0.673] | [0.384] | [0.727] | [0.394] | [0.623] |
| **SC** | **Trust** | 0.134*** | 0.0269* | 0.158*** | 0.0637*** | 0.162*** | 0.0493*** | 0.153*** | 0.0488*** |
|  |  | (-8.650) | (-1.720) | (-9.790) | (-3.880) | (-9.320) | (-2.800) | (-9.450) | (-2.940) |
|  |  | [1.143] | [1.027] | [1.171] | [1.066] | [1.154] | [1.044] | [1.165] | [1.050] |
|  | **Fair** | 0.102*** | 0.0643*** | 0.106*** | 0.0713*** | 0.115*** | 0.0902*** | 0.0886*** | 0.0899*** |
|  |  | (-8.180) | (-5.040) | (-8.020) | (-5.120) | (-9.320) | (-3.950) | (-6.610) | (-6.390) |
|  |  | [1.107] | [1.066] | [1.112] | [1.074] | [1.122] | [1.052] | [1.093] | [1.094] |
|  | **Support** | 0.000 | -0.030*** | -0.0101** | -0.0209*** | -0.003 | -0.0125** | -0.004 | -0.037*** |
|  |  | (-0.100) | (-6.03) | (-2.06) | (-3.98) | (-0.68) | (-2.49) | (-0.80) | (-7.38) |
|  |  | [1.000] | [0.972] | [0.990] | [0.979] | [0.997] | [0.988] | [0.996] | [0.963] |
|  | **Relations** | 0.011 | 0.000 | 0.040*** | 0.022** | 0.022** | 0.002 | 0.028*** | 0.018* |
|  |  | (-1.180) | (-0.010) | (-4.040) | (-2.130) | (-2.370) | (-0.170) | (-2.800) | (-1.740) |
|  |  | [1.011] | [1.000] | [1.041] | [1.023] | [1.022] | [1.002] | [1.029] | [1.019] |
|  | **Jiegousc** | 0.013 | -0.020** | 0.017** | -0.007 | 0.013** | -0.023** | 0.014* | -0.028** |
|  |  | (-1.630) | (-2.40) | (-2.040) | (-0.81) | (-1.510) | (-2.53) | (-1.650) | (-1.18) |
|  |  | [1.013] | [0.980] | [1.017] | [0.993] | [1.013] | [0.978] | [1.013] | [0.990] |
| **CV** | | Yes | Yes | Yes | Yes | Yes | Yes | Yes | Yes |
| **PDV** | | Yes | Yes | Yes | Yes | Yes | Yes | Yes | Yes |
| **Observations** | | 11，879 | 11，879 | 10，872 | 10，872 | 11，546 | 11，546 | 11，205 | 11，205 |
| **Prob>F** | | 0.000 | 0.000 | 0.000 | 0.000 | 0.000 | 0.000 | 0.000 | 0.000 |
| **Pseudo R^2^** | | 0.042 | 0.058 | 0.046 | 0.051 | 0.049 | 0.062 | 0.042 | 0.056 |

***Note:*** MH = Mental health, PH = Physical health, IIequality = Income inequality, SC = Social capital, CV = Control variable, PDV = Province dummy variablet.

**Table S6.** Results of regional heterogeneity test

| **Variable** | | **(1)** | **(2)** | **(3)** | **(4)** | **(5)** | **(6)** |
| --- | --- | --- | --- | --- | --- | --- | --- |
|  |  | **MH** | **PH** | **MH** | **PH** | **MH** | **PH** |
|  | | **Eastern region** | | **Central region** | | **Western region** | |
| **IIequality** | | -1.104*** | -0.529*** | -1.088*** | -0.352** | -0.693*** | -0.207* |
|  |  | (-11.27) | (-5.24) | (-7.12) | (-2.24) | (-5.71) | (-1.64) |
|  |  | [0.331] | [0.589] | [0.337] | [0.703] | [0.500] | [0.813] |
| **SC** | **Trust** | 0.148*** | 0.047*** | 0.123*** | 0.038 | 0.147*** | 0.0359* |
|  |  | (-9.280) | (-2.900) | (-4.870) | (-1.460) | (-7.410) | (-1.780) |
|  |  | [1.159] | [1.048] | [1.131] | [1.038] | [1.159] | [1.037] |
|  | **Fair** | 0.125*** | 0.093*** | 0.115*** | 0.055*** | 0.097*** | 0.042** |
|  |  | (-7.950) | (-6.730) | (-5.740) | (-2.680) | (-6.000) | (-2.550) |
|  |  | [1.110] | [1.098] | [1.122] | [1.057] | [1.101] | [1.043] |
|  | **Support** | -0.011** | -0.028*** | -0.002 | -0.030*** | 0.005 | -0.017*** |
|  |  | (-2.23) | (-5.51) | (-0.22) | (-3.88) | (-0.760) | (-2.63) |
|  |  | [0.989] | [0.973] | [0.998] | [0.971] | [1.005] | [0.983] |
|  | **Relations** | 0.048*** | 0.001 | 0.024* | 0.014 | -0.007 | 0.017 |
|  |  | (-4.750) | (-0.130) | (-1.640) | (-0.850) | (-0.60) | (-1.440 |
|  |  | [1.049] | [1.001] | [1.025] | [1.014] | [0.993] | [1.017] |
|  | **Jiegousc** | 0.0123* | -0.008* | 0.002* | -0.036** | 0.026** | -0.015 |
|  |  | (-1.650) | (-1.08) | (-0.130) | (-2.45) | (-2.180) | (-1.26) |
|  |  | [1.012] | [0.992] | [1.002] | [0.964] | [1.026] | [0.985] |
| **CV** | | Yes | Yes | Yes | Yes | Yes | Yes |
| **PDV** | | Yes | Yes | Yes | Yes | Yes | Yes |
| **Observations** | | 10952 | 10952 | 4926 | 4926 | 6873 | 6873 |
| **Prob>F** | | 0.000 | 0.000 | 0.000 | 0.000 | 0.000 | 0.000 |
| **Pseudo R^2^** | | 0.046 | 0.054 | 0.037 | 0.059 | 0.043 | 0.060 |

***Note*:** MH = Mental health, PH = Physical health, IIequality = Income inequality, SC = Social capital, CV = Control variable, PDV = Province dummy variablet.
